# A New Series of Indeno[1,2-*c*]pyrazoles as EGFR TK Inhibitors for NSCLC Therapy

**DOI:** 10.3390/molecules27020485

**Published:** 2022-01-13

**Authors:** Ahmet Özdemir, Halilibrahim Ciftci, Belgin Sever, Hiroshi Tateishi, Masami Otsuka, Mikako Fujita, Mehlika Dilek Altıntop

**Affiliations:** 1 Department of Pharmaceutical Chemistry, Faculty of Pharmacy, Anadolu University, Eskisehir 26470, Turkey; belginsever@anadolu.edu.tr; 2 Department of Drug Discovery, Science Farm Ltd., Kumamoto 862-0976, Japan; hiciftci@kumamoto-u.ac.jp (H.C.); motsuka@gpo.kumamoto-u.ac.jp (M.O.); 3 Medicinal and Biological Chemistry Science Farm Joint Research Laboratory, Faculty of Life Sciences, Kumamoto University, Kumamoto 862-0973, Japan; htateishi@kumamoto-u.ac.jp; 4 Department of Molecular Biology and Genetics, Koc University, Istanbul 34450, Turkey

**Keywords:** anticancer activity, apoptosis, epidermal growth factor receptor, indenopyrazoles, microwave-assisted synthesis, non-small cell lung cancer, tyrosine kinases

## Abstract

Non-small cell lung cancer (NSCLC) is the leading cause of cancer-related death throughout the world. Due to the shortcomings of traditional chemotherapy, targeted therapies have come into prominence for the management of NSCLC. In particular, epidermal growth factor receptor (EGFR) tyrosine kinase inhibitor (TKI) therapy has emerged as a first-line therapy for NSCLC patients with EGFR-activating mutations. In this context, new indenopyrazoles, which were prepared by an efficient microwave-assisted method, were subjected to in silico and in vitro assays to evaluate their potency as EGFR TK-targeted anti-NSCLC agents. Compound **4** was the most promising antitumor agent towards A549 human lung adenocarcinoma cells, with an IC_50_ value of 6.13 µM compared to erlotinib (IC_50_ = 19.67 µM). Based on its low cytotoxicity to peripheral blood mononuclear cells (PBMCs), it can be concluded that compound **4** exerts selective antitumor action. This compound also inhibited EGFR TK with an IC_50_ value of 17.58 µM compared to erlotinib (IC_50_ = 0.04 µM) and induced apoptosis (56.30%). Taking into account in silico and in vitro data, compound **4** stands out as a potential EGFR TKI for the treatment of NSCLC.

## 1. Introduction

Non-small cell lung cancer (NSCLC), which accounts for 85% of all lung cancers, represents the most common cause of cancer-related mortality worldwide [1,2], with a 5-year survival rate of less than 15% [3]. The best therapy option for NSCLC patients continues to be the complete surgical resection of the tumor [4]. However, most NSCLC patients are diagnosed at advanced or metastatic stages (stage III/IV), when surgery is no longer an option. In these cases, radiotherapy and chemotherapy are important therapeutic approaches for unresectable NSCLC [4,5,6,7].

Platinum-based chemotherapy is an existing Food and Drug Administration (FDA)-approved strategy in the management of several common cancer types, including NSCLC, and has many benefits in some cases [8,9]. Traditional chemotherapeutic agents eliminate not only rapidly dividing cancer cells but also normal cells, and therefore these drugs are highly toxic to all cells in the body [8,9,10]. Traditional cytotoxic chemotherapy also causes changes in the normal function of the cells, and correspondingly various side effects such as fatigue, anemia, alopecia, and gastrointestinal complications [8,9,10]. 

The drawbacks of traditional cytotoxic chemotherapy have raised the importance of targeted therapies acting on crucial signaling pathways and/or specific oncogenes deregulated in NSCLC [11]. One of the most promising targets is epidermal growth factor receptor (EGFR), a member of the ErbB/HER family of receptor tyrosine kinases (RTKs). EGFR plays a fundamental role in the regulation of cell proliferation, migration, differentiation, and survival [12,13], and the deregulation of EGFR leads to enhanced signaling activity that promotes tumor proliferation, invasion, angiogenesis, and metastasis [11]. EGFR is overexpressed in 40–89% of NSCLC cases and is mutated in 15–20% of NSCLC patients [11]. 

The discovery of EGFR-activating mutations in NSCLC and the success story of EGFR tyrosine kinase inhibitors (TKIs) have shifted the paradigm of cancer therapy from cytotoxic chemotherapy to targeted therapies [14]. Accordingly, EGFR TKI therapy (including erlotinib, gefitinib, and afatinib) has emerged as a first-line therapy for NSCLC patients with EGFR-activating mutations [14,15]. However, most patients inevitably develop drug resistance caused by the T790M mutation after almost 12 months of treatment [14,15,16]. In order to overcome the acquired resistance, next-generation EGFR TKIs have been approved for NSCLC therapy, and several promising candidates are currently undergoing clinical trials [14,15,16,17,18]. 

Indeno[1,2-*c*]pyrazole, which was first synthesized by Boyd in 1965, has emerged as a privileged scaffold found in many anticancer agents exerting their action through multiple mechanisms, such as the inhibition of EGFR TK, platelet-derived growth factor receptor (PDGFR) TK, vascular endothelial growth factor receptor-2 (VEGFR-2/KDR) TK, cyclin-dependent kinase (CDK), checkpoint kinase 1 (CHK1), Akt, hypoxia inducible factor 1 (HIF-1), and tubulin polymerization [19,20,21,22,23,24,25,26,27,28].

Prompted by the aforementioned findings related to indeno[1,2-*c*]pyrazoles exerting potent inhibitory effects on TKs (e.g., EGFR) involved in the pathogenesis of cancer, the microwave (MW)-assisted synthesis of new indeno[1,2-*c*]pyrazoles was performed efficiently, and in vitro studies were carried out to assess their potency as EGFR-targeted anti-NSCLC agents.

## 2. Results and Discussion

### 2.1. Chemistry

**Scheme 1:**
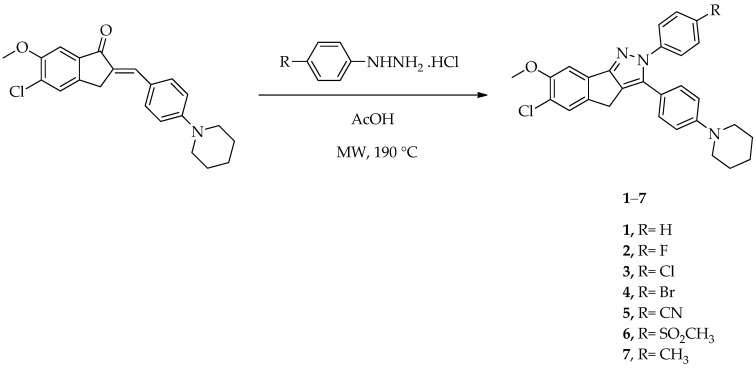
The synthetic route for the preparation of compounds **1**–**7**.

The synthesis of new indeno[1,2-*c*]pyrazoles (**1**–**7**) was carried out as depicted in Scheme 1. The Claisen–Schmidt condensation [29,30] of 5-chloro-6-methoxy-2,3-dihydro-1*H*-inden-1-one with 4-(piperidin-1-yl)benzaldehyde yielded 5-chloro-6-methoxy-2-[4-(piperidin-1-yl)benzylidene]-2,3-dihydro-1*H*-inden-1-one, which was previously synthesized by our research team [31], which underwent a MW-assisted cyclocondensation reaction with arylhydrazine hydrochloride and a few drops of acetic acid in ethanol, affording compounds **1**–**7**.

The Infrared (IR), ^1^H Nuclear Magnetic Resonance (NMR), ^13^C NMR, and High-Resolution Mass Spectrometry (HRMS) data were in agreement with the proposed structures of compounds **1**–**7**. In the IR spectra of compounds **1**–**7**, the absence of a C=O stretching band at 1684 cm^−1^ [31] confirmed that the ring closure reaction leading to the formation of the dihydroindenopyrazole scaffold occurred efficiently. The C=N and C=C stretching bands were located in the region of 1630–1450 cm^−1^. In the ^1^H NMR spectra of the compounds, the indene C_4_-C*H*_2_ protons were observed as a singlet at 3.32–3.75 ppm. In the ^1^H NMR spectra of compounds **1**–**7**, the protons attached to the C_3_, C_4_, and C_5_ carbons of the piperidine ring gave rise to a broad singlet at 1.57 or 1.65 ppm. The signals due to the protons attached to the C_2_ and C_6_ carbons of the piperidine ring appeared at 3.17–3.23 ppm as a broad singlet. All the other aliphatic and aromatic protons were observed as expected, e.g., Ar-OCH_3_ protons as a singlet at 3.93–4.01 ppm, and Ar-CH_3_ protons as a singlet at 2.39–3.10 ppm. The dihydroindenopyrazole C_4_-*C*H_2_ and O*C*H_3_ signals were observed at 27.63–30.10 and 52.33–59.69 ppm, respectively. The *C*_4_, *C*_3_, and *C*_5_, *C*_2_, and *C*_6_ carbons of the piperidine ring gave rise to the peaks at 22.68–25.53, 25.39–27.63, and 45.09–52.49 ppm in the ^13^C NMR spectra of compounds **1**–**7**. The formation of the dihydroindenopyrazole scaffold was also verified by the HRMS analysis of compounds **1**–**7**.

### 2.2. In Vitro Assays

Compounds **1**–**7** and erlotinib (positive control) were tested for their cytotoxic effects on A549 human lung adenocarcinoma cells using the MTT test, a tetrazolium-based cell viability assay. Compounds **1**, **4**, and **7** were found to be more effective than erlotinib. Compounds **1**, **4**, and **7** exerted their antitumor action towards A549 cells with IC_50_ values of 7.99, 6.13, and 8.67 µM, respectively, as compared to erlotinib (IC_50_ = 19.67 µM), an EGFR TKI currently used for NSCLC therapy (Table 1). Other compounds were found to be devoid of cytotoxic activity towards A549 cells (IC_50_ > 10 µM).

Among halogen substituents (F, Cl, Br), it can be concluded that the *p*-bromo group significantly enhances anti-NSCLC activity. *p*-Methyl substitution (−σ and +π effect) also led to marked anti-NSCLC activity, whereas *p*-methylsulfonyl and *p*-cyano substitutions (+σ and −π effect) resulted in the loss of anti-NSCLC activity.

Compounds **1**, **4**, and **7**, the most potent anti-NSCLC agents in this series, were investigated for their cytotoxic effects on K562 chronic myelogenous leukemia (CML) cells and peripheral blood mononuclear cells (PBMCs) to determine the selectivity of their antitumor action. Compounds **1**, **4**, and **7** showed marked anti-CML activity with IC_50_ values of 3.49, 2.65, and 2.76 µM, respectively (Table 1). On the other hand, compounds **1**, **4**, and **7** exerted no significant cytotoxicity towards PBMCs (normal cells). The selectivity index (SI) values of compounds **1**, **4**, and **7** (27.43, 7.38, and >36.23, respectively) were found to be higher than that of erlotinib (1.50), indicating that their anticancer effects were selective.

**Figure 1:**
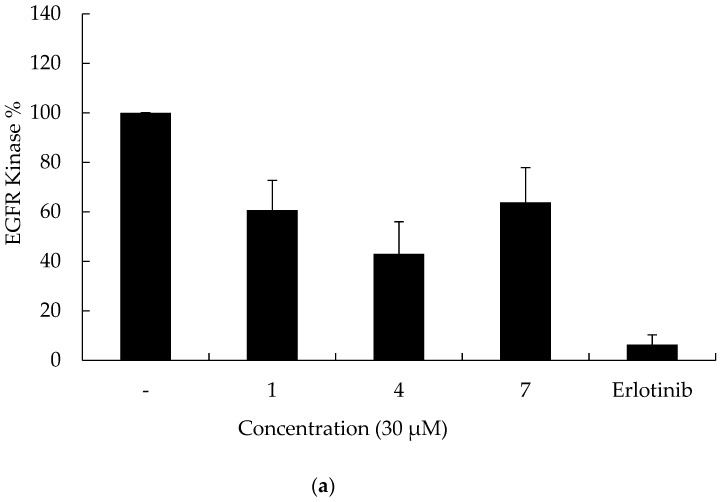

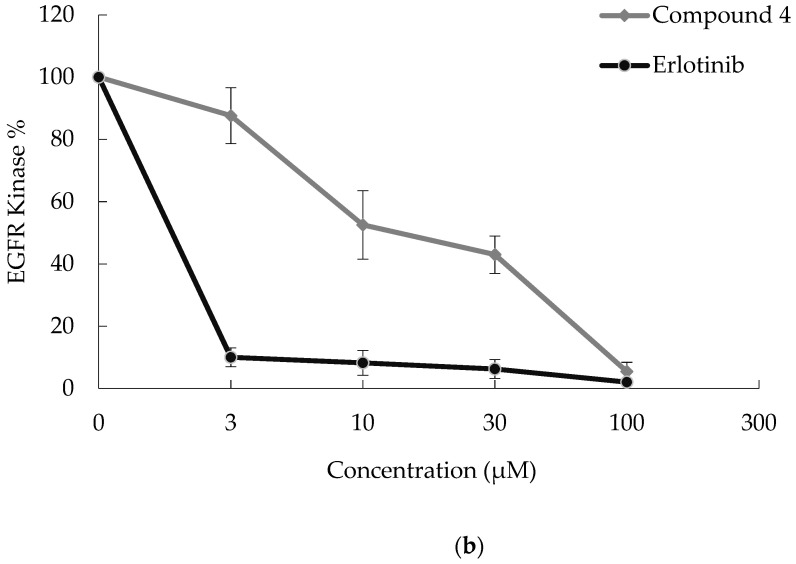
The inhibitory activity of the compounds on EGFR TK. (**a**) EGFR TK inhibition caused by compounds **1**, **4**, **7**, and erlotinib at 30 μM concentration. (**b**) The EGFR TK inhibition of compound **4** and erlotinib at different concentrations. All descriptive data were expressed as the Mean ± Standard Deviation (SD). All experiments were repeated three times.

Compounds **1**, **4**, and **7** were subjected to an in vitro mechanistic assay to determine whether their anti-NSCLC activities are related to EGFR TK inhibition or not. None of them inhibited EGFR TK as much as erlotinib (IC_50_ = 0.04 ± 0.01 µM). The most potent EGFR TKI in this series was found as compound **4** (IC_50_ = 17.58 ± 3.15 µM), followed by compounds **7** (IC_50_ = 46.66 ± 7.04 µM) and **1** (IC_50_ = 55.29 ± 8.68 µM) (Figure 1a). In particular, *p*-bromo substituent significantly enhanced EGFR TK inhibitory activity. According to Figure 1b, it can be concluded that compound **4** shows dose-dependent EGFR TK inhibitory potency.

**Figure 2:**
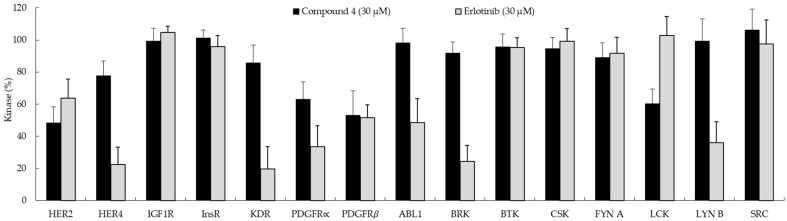
The inhibition of TKs by compound **4** and erlotinib at 30 μM concentration. All descriptive data were expressed as the Mean ± SD. All experiments were repeated three times.

Kinase selectivity profiling systems, TK-1 (EGFR, HER2, HER4, IGF1R, InsR, KDR, PDGFR-α, and PDGFR-β) and TK-2 (ABL1, BRK, BTK, CSK, FYN A, LCK, LYN B, and SRC), were used to identify the kinase selectivity profile of compound **4**. Compound **4** inhibited HER2 and LCK more significantly than erlotinib at 30 µM concentration (Figure 2). However, its inhibitory effects on HER2 and LCK were not as pronounced as its EGFR inhibitory activity. It is also important to note that the kinase selectivity profile of compound **4** is different from that of erlotinib.

**Figure 3:**
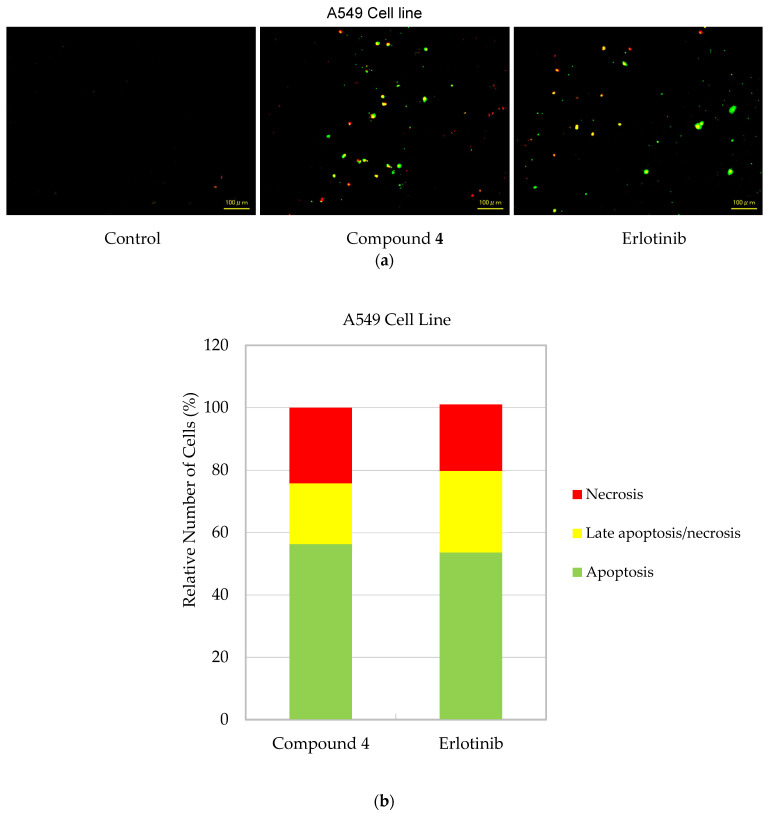
The apoptotic effects of compound **4** and erlotinib on A549 cells. (**a**) Coloring phenomenon of the A549 cell line following exposure to IC_50_ concentrations of compound **4** and erlotinib for 15 h. (**b**) The percentage of apoptotic (green), necrotic or late apoptotic (both green and red), and necrotic (red) cells was quantified by analyzing 100 randomly chosen stained cells in each experiment. All descriptive data were expressed as the Mean ± SD. All experiments were repeated three times.

In general, tumor cells lose their ability to undergo apoptosis, resulting in uncontrolled proliferation, and many antitumor agents trigger the induction of apoptosis to eliminate tumor cells [32]. Moreover, preclinical and clinical studies reveal that inhibition of EGFR, together with enhanced induction of apoptosis, may counter resistance to chemotherapy and radiotherapy [33]. In the current work, the pronounced EGFR TK-targeted anti-NSCLC activity of compound **4** prompted us to evaluate its apoptotic/necrotic effects using the annexin V/ethidium homodimer III staining assay. The results indicated that compound **4** induced apoptosis more than erlotinib, as shown in Figure 3. The percentages of A549 cells undergoing apoptosis exposed to compound **4** and erlotinib were found to be 56.30% and 53.60%, respectively.

### 2.3. In Silico Assays

#### 2.3.1. Molecular Docking Studies

**Figure 4:**
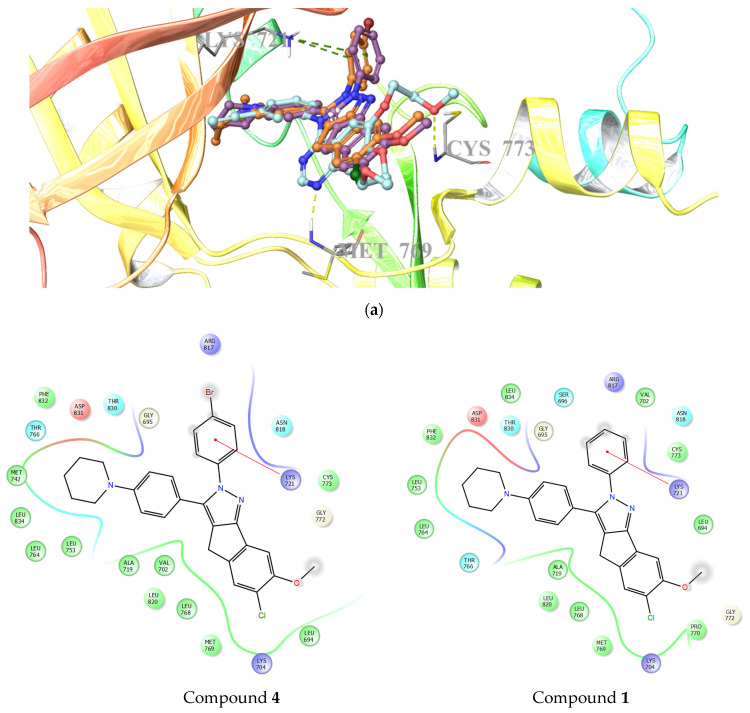

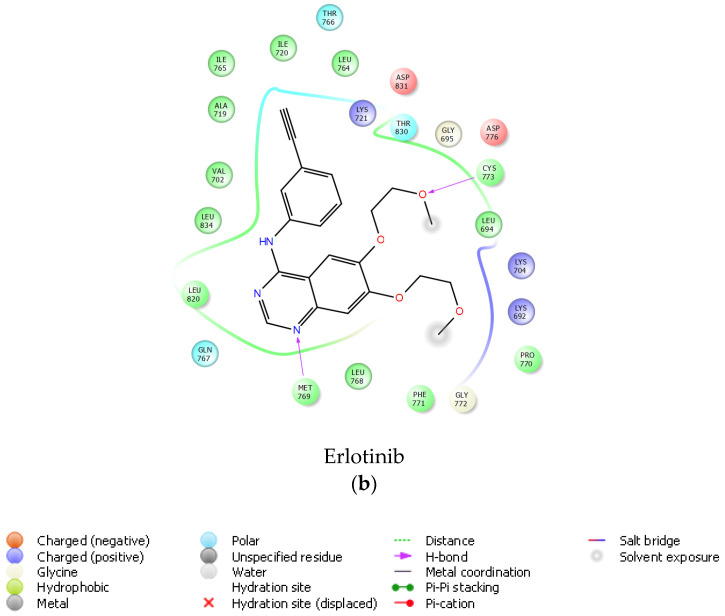
Docking poses of compound **4**, compound **1,** and erlotinib (**a**) (colored in orange, blue purple, and turquoise, respectively) (green dashes: π-cation interaction, yellow dashes: hydrogen bonding) and docking interactions of compound **4**, compound **1**, and erlotinib (**b**) in the ATP binding site of EGFR.

Molecular docking studies were carried out for compounds **4** and **1** to designate their binding profiles in the ATP binding site of EGFR compared to erlotinib (Figure 4a). The respective docking scores of compounds **4** and **1** were detected as −7.412 and −7.391 kcal/mol, which were similar to that of erlotinib (−8.895 kcal/mol). This outcome put emphasis on their high binding efficacy to the ATP binding site of EGFR. However, these compounds presented more different binding profiles than erlotinib. Compounds **4** and **1** formed π-cation interactions with Lys721 through its 4-bromophenyl and phenyl substitutions at the second position of 2,4-dihydroindeno[1,2-*c*]pyrazole, respectively, whereas they missed the important hydrogen bonding with Cys773 and Met769, which erlotinib established in the ATP binding site of EGFR (Figure 4b). This finding could enlighten the weaker EGFR inhibitory potency of compound **4** compared to erlotinib.

#### 2.3.2. In Silico Absorption, Distribution, Metabolism, and Excretion (ADME) Studies

Several crucial pharmacokinetic properties of compounds **1**–**7** were in silico ascertained. The QPlogBB and predicted central nervous system (CNS) activity values of compounds **1**–**7** were found within the specified ranges (Table 2), indicating that compounds **1**–**7** are able to cross the blood–brain barrier (BBB) and could be effective in CNS metastases in lung cancer. All synthesized compounds also exhibited a high percentage of human oral absorption, between 94.085% and 100%. These compounds violated only one parameter of Jorgensen’s rule of three. Besides, they also violated one parameter of Lipinski’s rule of five, apart from compounds **4** and **6** because both of them violated two parameters. Overall, these compounds were found to be orally bioavailable drug-like molecules for future studies.

## 3. Materials and Methods

### 3.1. Chemistry

All chemicals purchased from commercial suppliers were used without further purification. Melting points (M.p.) were detected on an Electrothermal IA9200 melting point device (Staffordshire, UK) and are uncorrected. IR spectra were recorded on an IRPrestige-21 Fourier Transform IR spectrophotometer (Shimadzu, Tokyo, Japan). NMR (^1^H and ^13^C) analyses were performed on an NMR spectrometer (Bruker, Billerica, MA, USA). HRMS spectra were recorded on a LCMS-IT-TOF system (Shimadzu, Kyoto, Japan). Thin-Layer Chromatography (TLC) was applied to monitor the progress of each chemical reaction and check the purity of each derivative.

#### General Procedure for the Synthesis of 6-Chloro-7-methoxy-2-aryl-3-[4-(piperidin-1-yl)phenyl]-2,4-dihydroindeno[1,2-*c*]pyrazoles (**1**–**7**)

A mixture of 5-chloro-6-methoxy-2-[4-(piperidin-1-yl)benzylidene]-2,3-dihydro-1*H*-inden-1-one (1 mmol) [31], arylhydrazine hydrochloride (3 mmol), and a few drops of acetic acid in ethanol (10 mL) was heated up to 190 °C within 15 min and kept at 190 °C for 20 min under MW irradiation in a reaction vial G30, sealed with a silicone septum and a snap cap with magnetic stirring at 600 rpm in an Anton Paar Monowave 400 Microwave Synthesis Reactor (Graz, Austria), equipped with a ruby thermometer. After the completion of the reaction, the temperature was decreased to 55 °C in the reactor. Then, the reaction mixture was further cooled to room temperature and the precipitate was collected by filtration. The dried product was purified.

*6-Chloro-7-methoxy-2-phenyl-3-[4-(piperidin-1-yl)phenyl]-2,4-dihydroindeno[1,2-c]pyrazole* (**1**), Dark beige powder. Yield: 89%. M.p. 153–155 °C. IR ν_max_ (cm^−1^): 3051 (aromatic C-H stretching), 2932, 2853 (aliphatic C-H stretching), 1630, 1599, 1572, 1566, 1547, 1530, 1512, 1503, 1493, 1483, 1468 (C=N and C=C stretching), 1443, 1433, 1402, 1391, 1383, 1366, 1356, 1294, 1258, 1236, 1169, 1126, 1109, 1055, 1028 (C-H bending, C-N, C-O stretching, and aromatic C-H in-plane bending), 916, 874, 862, 837, 816, 752, 691 (aromatic C-H out of plane bending). ^1^H NMR (300 MHz, DMSO-*d*_6_) δ (ppm): 1.65 (bs, 6H, piperidine C_3,4,5_-H), 3.23–3.27 (bs, 4H, piperidine C_2,6_-H), 3.70 (s, 2H, C_4_-H dihydroindenopyrazole), 4.01 (s, 3H, OCH_3_), 6.94–7.00 (m, 3H, aromatic protons), 7.06–7.24 (m, 2H, aromatic protons), 7.43 (d, *J* = 9.39 Hz, 2H, aromatic protons), 7.53 (d, *J* = 8.01 Hz, 2H, aromatic protons), 7.63 (d, *J* = 9.00 Hz, 2H, aromatic protons). ^13^C NMR (75 MHz, DMSO-*d*_6_) δ (ppm): 24.38 (CH_2_), 25.56 (2CH_2_), 29.76 (CH_2_), 48.98 (CH_2_), 49.47 (CH_2_), 55.29 (CH_3_), 102.88 (CH), 104.91 (CH), 106.74 (C), 112.21 (C), 112.99 (C), 115.30 (CH), 115.78 (CH), 116.21 (CH), 119.51 (2CH), 121.76 (2CH), 125.84 (C), 129.42 (2CH), 138.59 (C), 141.21 (C), 142.64 (C), 149.62 (C), 153.16 (C), 153.97 (C). HRMS (*m*/*z*): [M + H]^+^ calcd. for C_28_H_26_ClN_3_O: 456.1837. Found: 456.1816.

*6-Chloro-7-methoxy-2-(4-fluorophenyl)-3-[4-(piperidin-1-yl)phenyl]-2,4-dihydroindeno[1,2-c]pyrazole* (**2**), Brown powder. Yield: 34%. M.p. 188–190 °C. IR ν_max_ (cm^−1^): 3078 (aromatic C-H stretching), 2934, 2853 (aliphatic C-H stretching), 1605, 1508, 1468 (C=N and C=C stretching), 1435, 1400, 1385, 1366, 1294, 1250, 1234, 1223, 1167, 1119, 1103, 1053, 1022 (C-H bending, C-N, C-O stretching, and aromatic C-H in-plane bending), 974, 916, 862, 837, 818, 800, 777, 762, 743, 694 (aromatic C-H out of plane bending). ^1^H NMR (300 MHz, DMSO-*d*_6_) δ (ppm): 1.65 (bs, 6H, piperidine C_3,4,5_-H), 3.24–3.26 (bs, 4H, piperidine C_2,6_-H), 3.69 (s, 2H, C_4_-H dihydroindenopyrazole), 4.00 (s, 3H, OCH_3_), 6.96-7.00 (m, 3H, aromatic protons), 7.18 (d, *J* = 8.85 Hz, 1H, aromatic proton), 7.36–7.43 (m, 2H, aromatic protons), 7.50–7.55 (m, 2H, aromatic protons), 7.66–7.70 (m, 2H, aromatic protons). ^13^C NMR (75 MHz, DMSO-*d*_6_) δ (ppm): 24.43 (CH_2_), 25.55 (2CH_2_), 29.69 (CH_2_), 48.94 (CH_2_), 50.81 (CH_2_), 56.50 (CH_3_), 102.08 (CH), 103.11 (CH), 104.06 (CH), 113.84 (C), 115.28 (2CH), 121.31 (C), 126.10 (C), 127.03 (d, *J* = 18.38 Hz, 2CH), 128.04 (d, *J* = 15.29 Hz, CH), 129.57 (2CH), 131.45 (C), 137.77 (C), 139.23 (C), 142.28 (C), 149.31 (C), 149.77 (C), 152.58 (C), 153.63 (C). HRMS (*m*/*z*): [M + H]^+^ calcd. for C_28_H_25_ClFN_3_O: 474.1743. Found: 474.1726.

*6-Chloro-7-methoxy-2-(4-chlorophenyl)-3-[4-(piperidin-1-yl)phenyl]-2,4-dihydroindeno[1,2-c]pyrazole* (**3**), Dark brown powder. Yield: 56%. M.p. 103–106 °C. IR ν_max_ (cm^−1^): 3028 (aromatic C-H stretching), 2932, 2851 (aliphatic C-H stretching), 1599, 1487, 1470 (C=N and C=C stretching), 1433, 1385, 1360, 1294, 1260, 1236, 1167, 1126, 1092, 1051, 1024, 1011 (C-H bending, C-N, C-O stretching, and aromatic C-H in-plane bending), 972, 918, 895, 866, 824, 756, 745, 696 (aromatic C-H out of plane bending). ^1^H NMR (300 MHz, DMSO-*d*_6_) δ (ppm): 1.57 (bs, 6H, piperidine C_3,4,5_-H), 3.19–3.21 (bs, 4H, piperidine C_2,6_-H), 3.70 (s, 2H, C_4_-H dihydroindenopyrazole), 3.95 (s, 3H, OCH_3_), 6.93 (d, *J* = 8.97 Hz, 2H, aromatic protons), 7.08–7.15 (m, 2H, aromatic protons), 7.38 (d, *J* = 8.46 Hz, 2H, aromatic protons), 7.47–7.53 (m, 2H, aromatic protons), 7.61–7.64 (m, 2H, aromatic protons). ^13^C NMR (75 MHz, DMSO-*d*_6_) δ (ppm): 24.32 (CH_2_), 25.55 (2CH_2_), 29.76 (CH_2_), 48.92 (CH_2_), 52.49 (CH_2_), 56.97 (CH_3_), 114.38 (C), 115.32 (2CH), 118.41 (C), 120.39 (C), 121.43 (C), 127.27 (4CH), 129.52 (2CH), 129.64 (2CH), 131.45 (C), 132.17 (C), 139.66 (C), 140.12 (C), 141.09 (C), 148.66 (C), 150.33 (C). HRMS (*m*/*z*): [M + H]^+^ calcd. for C_28_H_25_Cl_2_N_3_O: 490.1447. Found: 490.1432.

*6-Chloro-7-methoxy-2-(4-bromophenyl)-3-[4-(piperidin-1-yl)phenyl]-2,4-dihydroindeno[1,2-c]pyrazole* (**4**), Camel powder. Yield: 80%. M.p. 125–126 °C. IR ν_max_ (cm^−1^): 3063 (aromatic C-H stretching), 2934, 2851 (aliphatic C-H stretching), 1601, 1485, 1468 (C=N and C=C stretching), 1433, 1385, 1360, 1294, 1261, 1238, 1198, 1167, 1126, 1055, 1024, 1009 (C-H bending, C-N, C-O stretching, and aromatic C-H in-plane bending), 970, 918, 891, 862, 827, 754, 745, 696 (aromatic C-H out of plane bending) (Supplementary Materials Figure S1). ^1^H NMR (300 MHz, DMSO-*d*_6_) δ (ppm): 1.57 (bs, 6H, piperidine C_3,4,5_-H), 3.19–3.21 (bs, 4H, piperidine C_2,6_-H), 3.64 (s, 2H, C_4_-H dihydroindenopyrazole), 3.94 (s, 3H, OCH_3_), 6.93 (d, *J* = 8.97 Hz, 1H, aromatic proton), 7.14 (d, *J* = 8.85 Hz, 1H, aromatic proton), 7.19–7.23 (m, 1H, aromatic proton), 7.31–7.37 (m, 3H, aromatic protons), 7.43–7.50 (m, 2H, aromatic protons), 7.61–7.66 (m, 2H, aromatic protons) (Figure S2). ^13^C NMR (75 MHz, DMSO-*d*_6_) δ (ppm): 22.68 (CH_2_), 25.62 (2CH_2_), 30.10 (CH_2_), 46.75 (CH_2_), 48.90 (CH_2_), 59.69 (CH_3_), 104.21 (CH), 108.25 (CH), 109.28 (CH), 115.40 (2CH), 118.32 (2C), 126.25 (C), 127.59 (2CH), 129.73 (2CH), 132.08 (C), 133.64 (CH), 138.13 (2C), 139.30 (2C), 148.42 (C), 150.32 (C), 154.38 (C) (Figure S3). HRMS (*m*/*z*): [M + H]^+^ calcd. for C_28_H_25_BrClN_3_O: 534.0942. Found: 534.0926 (Figure S4).

*6-Chloro-7-methoxy-2-(4-cyanophenyl)-3-[4-(piperidin-1-yl)phenyl]-2,4-dihydroindeno[1,2-c]pyrazole* (**5**), Beige powder. Yield: 47%. M.p. 175–176 °C. IR ν_max_ (cm^−1^): 3080 (aromatic C-H stretching), 2945, 2934, 2862 (aliphatic C-H stretching), 2214 (C≡N stretching), 1603, 1531, 1512, 1476 (C=N and C=C stretching), 1449, 1422, 1389, 1348, 1335, 1327, 1314, 1296, 1267, 1254, 1236, 1223, 1192, 1167, 1126, 1096, 1055, 1024, 1007 (C-H bending, C-N, C-O stretching, and aromatic C-H in-plane bending), 962, 945, 916, 866, 833, 818, 810, 768, 716, 656 (aromatic C-H out of plane bending). ^1^H NMR (300 MHz, DMSO-*d*_6_) δ (ppm): 1.58 (bs, 6H, piperidine C_3,4,5_-H), 3.23 (bs, 4H, piperidine C_2,6_-H), 3.32 (s, 2H, C_4_-H dihydroindenopyrazole), 3.92 (s, 3H, OCH_3_), 6.94 (d, *J* = 8.88 Hz, 1H, aromatic proton), 7.01 (d, *J* = 8.97 Hz, 1H, aromatic proton), 7.08 (d, *J* = 8.70 Hz, 1H, aromatic proton), 7.24 (d, *J* = 8.85 Hz, 1H, aromatic proton), 7.30–7.34 (m, 2H, aromatic protons), 7.45 (s, 1H, aromatic proton), 7.50–7.63 (m, 3H, aromatic protons). ^13^C NMR (75 MHz, DMSO-*d*_6_) δ (ppm): 24.37 (CH_2_), 25.49 (2CH_2_), 27.82 (CH_2_), 49.02 (2CH_2_), 56.77 (CH_3_), 99.20 (C), 103.90 (CH), 112.17 (CH), 113.30 (2CH), 114.87 (CH), 115.44 (CH), 120.68 (C), 123.90 (C), 127.53 (2CH), 130.76 (CH), 133.65 (C), 133.95 (CH), 138.94 (C), 140.57 (C), 140.96 (C), 141.35 (C), 149.33 (C), 149.69 (C), 153.72 (C), 154.45 (C). HRMS (*m*/*z*): [M + H]^+^ calcd. for C_29_H_25_ClN_4_O: 481.1790. Found: 481.1781.

*6-Chloro-7-methoxy-2-(4-(methylsulfonyl)phenyl)-3-[4-(piperidin-1-yl)phenyl]-2,4-dihydroindeno[1,2-c]pyrazole* (**6**), Brown powder. Yield: 71%. M.p. 116–117 °C. IR ν_max_ (cm^−1^): 3013 (aromatic C-H stretching), 2928, 2851 (aliphatic C-H stretching), 1591, 1510, 1472, 1452 (C=N and C=C stretching), 1414, 1385, 1290, 1231, 1180, 1126, 1090, 1055, 1024, 1003 (C-H bending, SO_2_, C-N, C-O stretching, and aromatic C-H in-plane bending), 955, 918, 829, 768, 710, 702, 687 (aromatic C-H out of plane bending). ^1^H NMR (300 MHz, DMSO-*d*_6_) δ (ppm): 1.59 (bs, 6H, piperidine C_3,4,5_-H), 3.10 (s, 3H, CH_3_), 3.22–3.23 (bs, 4H, piperidine C_2,6_-H), 3.75 (s, 2H, C_4_-H dihydroindenopyrazole), 3.95 (s, 3H, OCH_3_), 6.95 (d, *J* = 8.97 Hz, 1H, aromatic proton), 7.02 (d, *J* = 8.97 Hz, 1H, aromatic proton), 7.29–7.36 (m, 2H, aromatic protons), 7.39–7.48 (m, 1H, aromatic proton), 7.50–7.53 (m, 1H, aromatic proton), 7.57 (d, *J* = 8.85 Hz, 1H, aromatic proton), 7.63–7.73 (m, 3H, aromatic protons). ^13^C NMR (75 MHz, DMSO-*d*_6_) δ (ppm): 25.45 (CH_2_), 25.52 (2CH_2_), 29.43 (CH_2_), 44.76 (CH_3_), 48.90 (2CH_2_), 52.33 (CH_3_), 111.54 (CH), 113.27 (2CH), 114.99 (2CH), 115.46 (2CH), 123.35 (C), 128.04 (CH), 129.32 (2CH), 135.22 (C), 138.41 (C), 141.31 (C), 143.65 (C), 147.70 (C), 152.49 (C), 154.05 (C), 158.63 (2C), 160.82 (C). HRMS (*m*/*z*): [M + H]^+^ calcd. for C_29_H_28_ClN_3_O_3_S: 534.1613. Found: 534.1602.

*6-Chloro-7-methoxy-2-(4-methylphenyl)-3-[4-(piperidin-1-yl)phenyl]-2,4-dihydroindeno[1,2-c]pyrazole* (**7**), Camel powder. Yield: 37%. M.p. 123–124 °C. IR ν_max_ (cm^−1^): 3015 (aromatic C-H stretching), 2932, 2853 (aliphatic C-H stretching), 1609, 1514, 1497, 1466, 1450 (C=N and C=C stretching), 1431, 1383, 1360, 1294, 1261, 1238, 1180, 1126, 1109, 1055, 1024 (C-H bending, C-N, C-O stretching, and aromatic C-H in-plane bending), 974, 918, 862, 820, 800, 762, 743, 694 (aromatic C-H out of plane bending). ^1^H NMR (300 MHz, DMSO-*d*_6_) δ (ppm): 1.58 (bs, 6H, piperidine C_3,4,5_-H), 2.39 (s, 3H, CH_3_), 3.17–3.19 (bs, 4H, piperidine C_2,6_-H), 3.60 (s, 2H, C_4_-H dihydroindenopyrazole), 3.93 (s, 3H, OCH_3_), 6.88–6.95 (m, 3H, aromatic protons), 7.12 (d, *J* = 8.76 Hz, 1H, aromatic proton), 7.25 (s, 2H, aromatic protons), 7.32–7.36 (m, 2H, aromatic protons), 7.48 (s, 1H, aromatic proton), 7.56 (s, 1H, aromatic proton). ^13^C NMR (75 MHz, DMSO-*d*_6_) δ (ppm): 21.17 (CH_3_), 25.53 (2CH_2_), 27.63 (2CH_2_), 45.09 (CH_2_), 49.08 (CH_2_), 59.14 (CH_3_), 112.58 (2CH), 115.26 (4CH), 124.23 (C), 125.61 (CH), 128.72 (CH), 129.71 (2CH), 132.17 (C), 137.88 (C), 140.30 (2C), 140.93 (C), 147.22 (C), 148.66 (C), 150.33 (C), 155.59 (C), 156.85 (C). HRMS (*m*/*z*): [M + H]^+^ calcd. for C_29_H_28_ClN_3_O: 470.1994. Found: 470.1981.

### 3.2. Biochemistry

#### 3.2.1. Cell Culture and Drug Treatments

K562 cells and PBMCs (Precision Bioservices, Frederic, MD, USA) were cultured in RPMI 1640 (Wako Pure Chemical Industries, Osaka, Japan), while A549 cells were cultured in DMEM/Ham’s F-12 (Wako Pure Chemical Industries, Osaka, Japan). All cells were supplemented with 10% fetal bovine serum (FBS) (Sigma Aldrich, MO, USA) and 89 μg/mL of streptomycin (Meiji Seika Pharma, Tokyo, Japan) at 37 °C in a humid atmosphere and 5% CO_2_. In experiments, K562 cells and PBMCs were cultured in 24-well and 96-well plates (Asahi Glass Co., Chiba, Japan) at 4 × 10^4^ and 1 × 10^6^ cells/mL concentrations, respectively, for 48 h. A549 cells were plated (24-well plate) at 1 × 10^4^ cells/mL and incubated for 72 h (the optimal cell number was determined in our previous studies) [34,35,36]. The stock solutions of the compounds and erlotinib in concentrations ranging from 1 µM to 10 mM were prepared in DMSO (Wako Pure Chemical Industries, Osaka, Japan) and further diluted with fresh culture medium. The final DMSO concentration was adjusted as 1%, which had no effect on the cell viability [37].

#### 3.2.2. Cell Viability Assay

MTT (Dojindo Molecular Technologies, Kumamoto, Japan) was used to evaluate the cytotoxic effects of the compounds and erlotinib on A549 and K562 cells, and PBMCs, based on the previously described procedures in the literature [37,38]. After cells were exposed to various concentrations (0.1–100 µM) of the compounds for 48 and 72 h at 37 °C, MTT solution was applied and cells were incubated for an additional 4 h. Then, supernatants were discarded, and the formazan crystals were solubilized with a 100 μL DMSO addition. The absorbance of the solution was measured using an Infinite M1000 microplate reader (Tecan, Grödig, Austria) at a wavelength of 570 and 630 nm. All experiments were conducted three times and IC_50_ values were specified as the drug concentrations, decreasing absorbance to 50% of control values [39].

#### 3.2.3. Kinase Inhibitory Activity

Manufacturer′s instructions (Promega Corporation, Madison, WI, USA) were applied for the kinase profiling assay protocol (TK-1 and TK-2) with small changes [40,41,42]. According to this protocol, the kinase stocks in the kinase strips and their substrate stocks in the substrate strips were diluted with 95 µL of 2.5× kinase buffer and 15 µL of 100 µM ATP solutions, respectively. The kinase working stocks (2 µL), 2 µL of the ATP/substrate working stocks, and 1 µL of the tested compound solution (3–100 µM) and erlotinib solution (0.01–100 µM) or 5% DMSO solution in the 384-well plate were used for the kinase reactions. Following the 4 h of incubation at room temperature, the activity of kinases was screened by means of the ADP-Glo Kinase Assay (Promega Corporation, Madison, WI, USA) based on the manufacturer′s protocol. The Infinite M1000 microplate reader (Tecan, Grödig, Austria) was used to measure the kinase inhibitory effects of the compounds in a dose-response manner. Besides, the IC_50_ values of the tested compounds were determined using ImageJ software.

#### 3.2.4. Detection of Cell Death

A549 cells (1 × 10^4^ cells/well) were incubated in each well of a 24-well plate with the most potent compounds in this series at IC_50_ concentrations for 15 h. Then, an apoptotic/necrotic cell detection kit (PromoKine, Heidelberg, Germany) was used according to manufacturer′s instructions with some modifications [43,44]. Briefly, the cells were washed twice with 1× binding buffer, a staining solution containing 50 μL of 1× binding buffer, 5 μL of FITC-Annexin V solution, and 5 μL of ethidium homodimer III solution, and treated for 30 min at room temperature in a protected-light environment. After washing of cells in 1× binding buffer, cells were analyzed under a Biorevo BZ-9000 all-in-one fluorescence microscope (Keyence, Osaka, Japan). The number of apoptotic cells, late apoptotic or necrotic cells, and necrotic cells was quantified as previously described [45].

### 3.3. Molecular Docking

The X-ray crystallographic structure of EGFR (supplied from the PDB server (PDB code: 4HJO) [46]) was optimized in the protein preparation module. After that, compound **4**, compound **1**, and erlotinib were optimized with energy minimization in the ligand preparation module. Molecular docking simulations were generated for optimized EGFR and ligands with Grid Generation and Glide/XP protocols (Schrödinger Release 2016-2: Schrödinger, LLC, New York, NY, USA).

### 3.4. In Silico ADME Prediction

*In silico* pharmacokinetic profiles of compounds **1**–**7** were determined using the QikProp module of Schrödinger software (Schrödinger Release 2016-2: QikProp, Schrödinger, LLC, New York, NY, USA).

## 4. Conclusions

In this paper, a MW-assisted technique was employed to efficiently synthesize a new series of 2,4-dihydroindeno[1,2-*c*]pyrazoles. The MTT assay was conducted to determine their potency as anti-NSCLC agents. Compounds **1**, **4**, and **7** were found to be more potent than erlotinib on A549 cells. These derivatives were also evaluated for their cytotoxic features on K562 CML cells and PBMCs to assess their selectivity. Based on the SI values of compounds **1**, **4**, and **7**, their anticancer activities were selective. The EGFR TK inhibitory effects of compounds **1**, **4**, and **7** were also investigated. Among them, *p*-bromo-substituted compound **4** was the most potent EGFR TKI with an IC_50_ value of 17.58 µM. In order to gain a better insight into its kinase selectivity profile, compound **4** was also tested for its inhibitory effects on HER2, HER4, IGF1R, InsR, KDR, PDGFR-α, PDGFR-β, ABL1, BRK, BTK, CSK, FYN A, LCK, LYN B, and SRC. Compound **4** showed more potent inhibitory effects on HER2 and LCK enzymes than erlotinib. According to in vitro kinase profiling assays, it can be concluded that the kinase selectivity profiles of compound **4** and erlotinib are different. Moreover, the annexin V/ethidium homodimer III staining method was applied to determine the effects of compound **4** and erlotinib on apoptosis. Compound **4** induced apoptosis more than erlotinib. Taking into account all in vitro data, compound **4** exerts selective and potent anti-NSCLC activity through the inhibition of EGFR TK, together with the induction of apoptosis, which may counter resistance to chemotherapy and radiotherapy. Molecular docking studies revealed that compound **4** displayed high binding affinity to the ATP binding site of EGFR, similar to erlotinib, but with a more distinct interaction profile. In silico ADME data of compound **4** pointed out its potential as a potential orally bioavailable anti-NSCLC agent endowed with favorable drug-like features. This work, comprising of a combination of biochemical and computational approaches, could represent a rational guideline for further structural modifications of 2,4-dihydroindeno[1,2-*c*]pyrazoles to generate a new class of EGFR TKIs for NSCLC therapy.

## Figures and Tables

**Table 1:** The cytotoxic effects of the compounds on A549 and K562 cells, and PBMCs.

Compound	R	IC_50_ (µM)			SI ^1^
		A549 Cells	K562 Cells	PBMCs	
**1**	H	7.99 ± 1.84	3.49 ± 0.92	95.72 ± 11.68	27.43
**2**	F	>10			
**3**	Cl	>10			
**4**	Br	6.13 ± 1.42	2.65 ± 0.75	19.56 ± 4.25	7.38
**5**	CN	>10			
**6**	SO_2_CH_3_	>10			
**7**	CH_3_	8.67 ± 2.18	2.76 ± 0.83	>100	>36.23
**Erlotinib**	-	19.67 ± 3.15	29.62 ± 5.26	44.33 ± 7.84	1.50

^1^ SI = IC_50_ for PBMCs/IC_50_ for K562 cells.

**Table 2:** Predicted ADME properties of compounds **1**–**7**.

Compound	QPlogBB * (−3–1.2)	CNS ** (−2 to 2)	Human Oral Absorption% ***	Rule of Five ****	Rule of Three *****
**1**	0.478	2	100	1	1
**2**	0.592	2	100	1	1
**3**	0.650	2	100	1	1
**4**	0.663	2	100	2	1
**5**	−0.388	0	100	1	1
**6**	−0.495	0	94.085	2	1
**7**	0.472	2	100	1	1

* QPlogBB: Brain/blood partition coefficient; ** CNS: Predicted central nervous system activity; *** Human Oral Absorption%: Predicted human oral absorption on a 0% to 100% scale. The prediction is based on a quantitative multiple linear regression model. This property usually correlates well with human oral absorption, as both measure the same property (>80% is high, <25% is poor); **** **Rule of Five:** Number of violations of Lipinski’s rule of five. The rules are: **mol_MW** (molecular weight of the molecule) < 500, **QPlogPo/w** (predicted octanol/water partition coefficient) < 5, **donorHB** (hydrogen-bond donor atoms) ≤ 5, and **accptHB** (hydrogen-bond acceptor atoms) ≤ 10. Compounds that provide these rules are considered drug-like (the “five” refers to the limits, which are multiples of 5); ***** **Rule of Three:** Number of violations of Jorgensen’s rule of three. The three rules are: **QPlogS** (predicted aqueous solubility) > –5.7, **QPPCaco** (predicted apparent Caco-2 cell permeability in nm/s) > 22 nm/s, and # **Primary Metabolites** < 7. Compounds with fewer (and preferably no) violations of these rules are more likely to be orally available (Schrödinger Release 2016-2: Schrödinger, LLC, New York, NY, USA).

## Data Availability

Not applicable.

## Supplementary Materials

The following are available online, Figure S1: The IR spectrum of compound **4**; Figure S2: The ^1^H NMR spectrum of compound **4**; Figure S3: The ^13^C NMR spectrum of compound **4**; Figure S4: The HRMS spectrum of compound **4**.

## Sample Availability

Samples of compounds **1**–**7** are available from the authors.
