# Supplementary material for: A New Series of Indeno[1,2-c]pyrazoles as EGFR TK Inhibitors for NSCLC Therapy
Source: Molecules. 2022 Jan 13;27(2):485. doi: 10.3390/molecules27020485 (PMC8778314; doi:10.3390/molecules27020485)
Supplement: Supplementary file 1 [file molecules-27-00485-s001.zip › molecules-1520018-supplementary.pdf]

Supplementary Material

A New Series of Indeno[1,2-*c*]pyrazoles as EGFR TK Inhibitors for NSCLC Therapy

Ahmet Özdemir [1,*], Halilibrahim Ciftci [2,3,4], Belgin Sever [1,3], Hiroshi Tateishi [3], Masami Otsuka [2,3], Mikako Fujita [3,*] and Mehlika Dilek Altıntop [1,*]

[1] Department of Pharmaceutical Chemistry, Faculty of Pharmacy, Anadolu University, Eskisehir 26470, Turkey; belginsever@anadolu.edu.tr

[2] Department of Drug Discovery, Science Farm Ltd., Kumamoto 862-0976, Japan; hiciftci@kumamoto-u.ac.jp (H.C.); motsuka@gpo.kumamoto-u.ac.jp (M.O.)

[3] Medicinal and Biological Chemistry Science Farm Joint Research Laboratory, Faculty of Life Sciences, Kumamoto University, Kumamoto 862-0973, Japan; htateishi@kumamoto-u.ac.jp

[4] Department of Molecular Biology and Genetics, Koc University, Istanbul 34450, Turkey

* Correspondence: ahmeto@anadolu.edu.tr (A.Ö.); mfujita@kumamoto-u.ac.jp (M.F.); mdaltintop@anadolu.edu.tr (M.D.A.); Tel.: +90-222-335-0580 (ext. 3780) (A.Ö.); +81-96-371-4622 (M.F.); +90-222-335-0580 (ext. 3807) (M.D.A.)

Figure S1. The IR spectrum of compound **4**

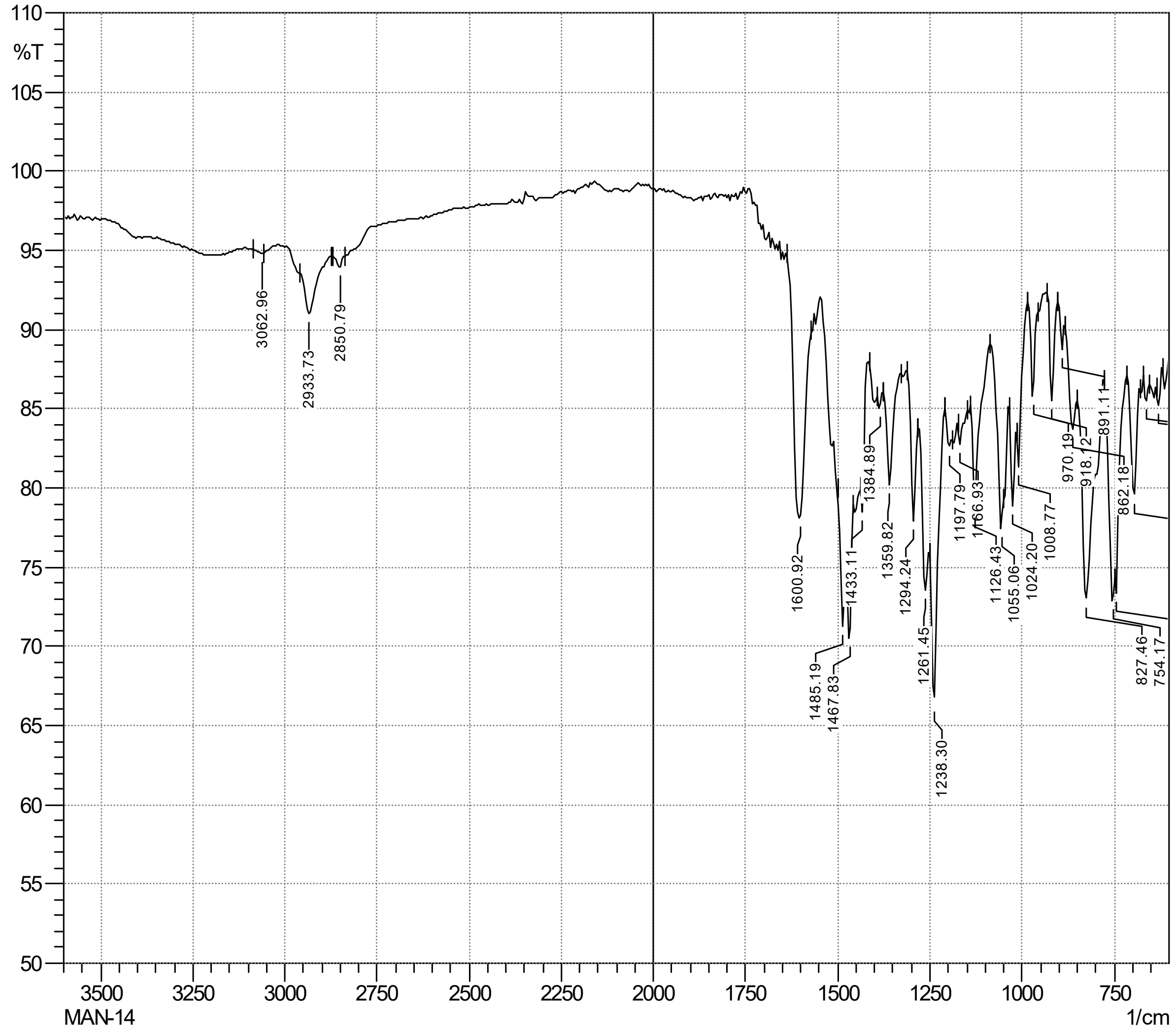

Figure S2. The ^{1}H NMR spectrum of compound **4**

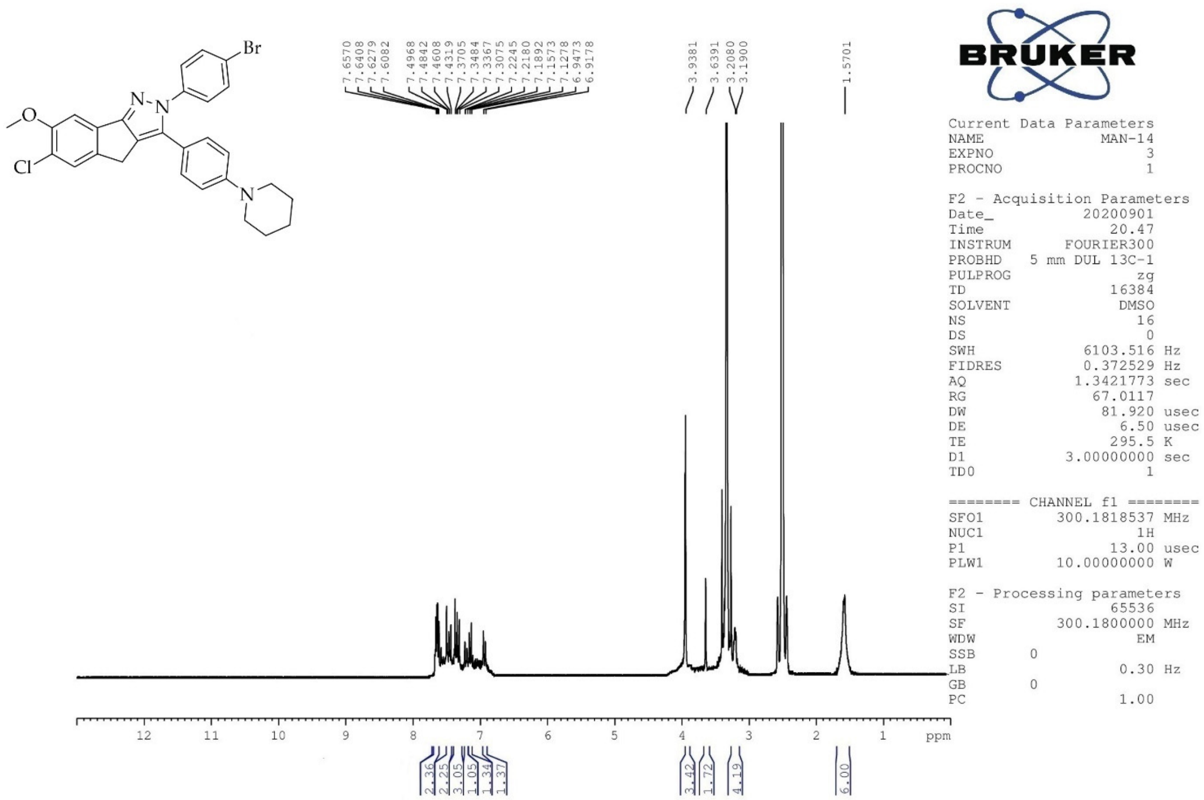

Figure S3. The ^{13}C NMR spectrum of compound **4**

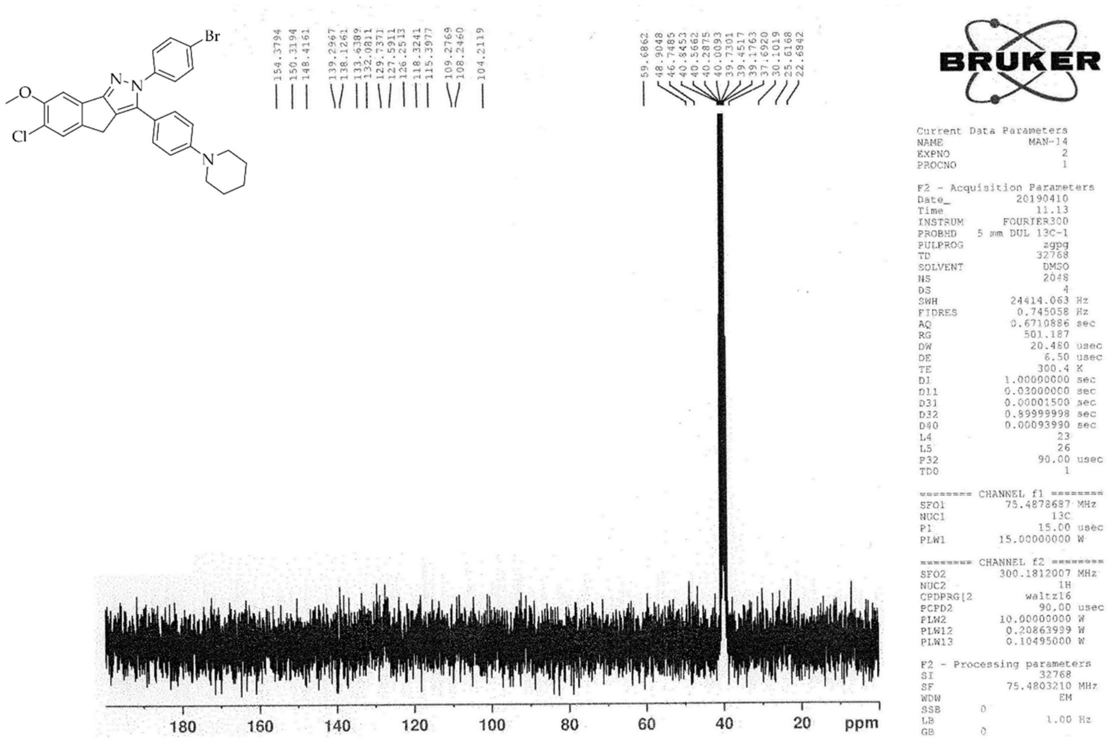

Figure S4. The HRMS spectrum of compound **4**

Formula Predictor Report - MAN-14_8.lcd

Page 1 of 1

Data File: C:\LabSolutions\Data\Analiz\AOzdemir\MAN-14_8.lcd

Elmt	Val.	Min	Max	Elmt	Val.	Min	Max	Elmt	Val.	Min	Max	Elmt	Val.	Min	Max	Use Adduct
H	1	9	35	O	2	0	5	S	2	0	0	Ru	2	0	0	H
C	4	26	35	F	1	0	0	Cl	1	1	4	Pd	2	0	0	
N	3	3	5	P	3	0	0	Br	1	1	1	I	3	0	0	

Error Margin (ppm): 5
HC Ratio: unlimited
Max Isotopes: 3
MSn Iso RI (%): 10.00

DBE Range: 5.0 - 20.0
Apply N Rule: yes
Isotope RI (%): 1.00
MSn Logic Mode: AND

Electron Ions: both
Use MSn Info: yes
Isotope Res: 9000
Max Results: 100

Event#: 1 MS(E+) Ret. Time : 7.093 -> 7.280 Scan# : 1065 -> 1093

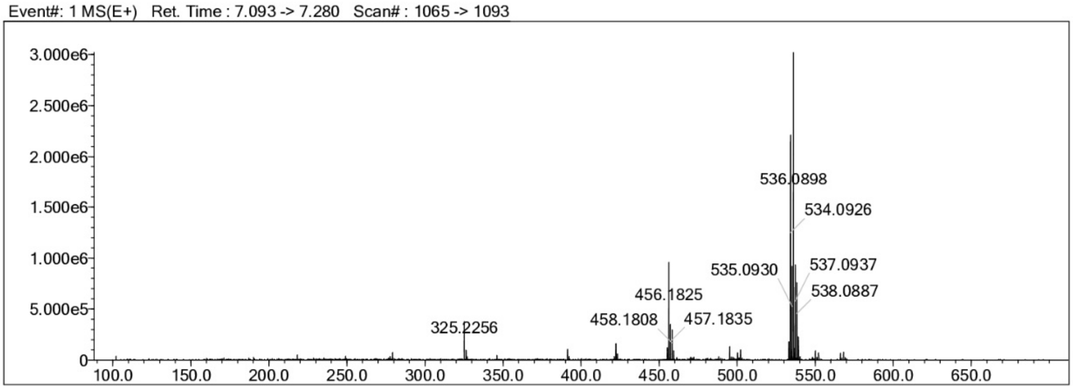

Measured region for 534.0926 m/z

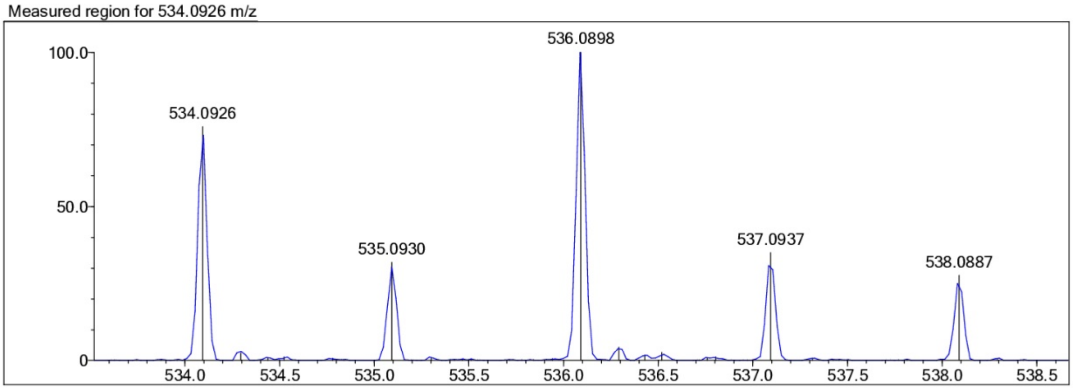

C28 H25 N3 O Cl Br [M+H]+ : Predicted region for 534.0942 m/z

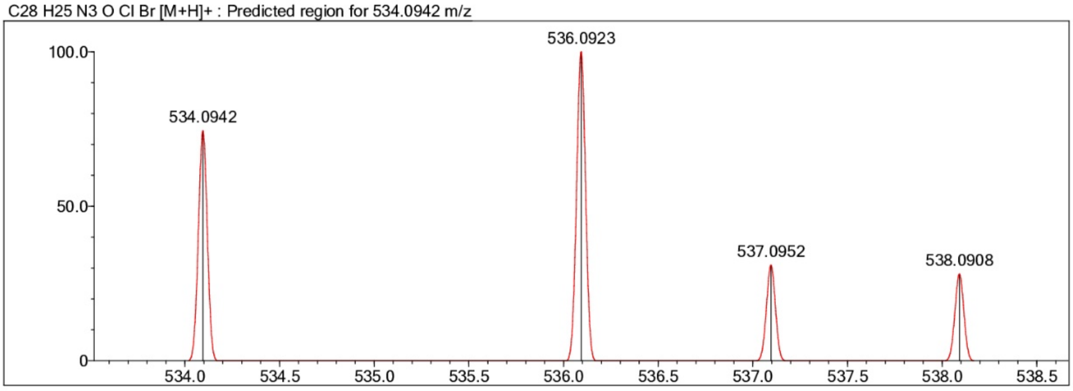

Rank	Score	Formula (M)	Ion	Meas. m/z	Pred. m/z	Df. (mDa)	Df. (ppm)	Iso	DBE
1	72.79	C28 H25 N3 O Cl Br	[M+H]+	534.0926	534.0942	-1.6	-3.00	76.62	17.0
